# Cancer Incidence of 2,4-D Production Workers

**DOI:** 10.3390/ijerph8093579

**Published:** 2011-09-02

**Authors:** Carol Burns, Kenneth Bodner, Gerard Swaen, James Collins, Kathy Beard, Marcia Lee

**Affiliations:** 1 The Dow Chemical Company, Epidemiology, 1803 Building, Midland, MI 48674, USA; E-Mails: kmbodner@dow.com (K.B.); jjcollins@dow.com (J.C.); 2 Dow Benelux BV, Gaspeldoorn 24, Maastricht, Limburg, 6226WR, The Netherlands; E-Mail: gswaen@dow.com; 3 The Dow Chemical Company, Industrial Hygiene, 1803 Building, Midland, MI 48674, USA; E-Mail: kkbeard@dow.com; 4 The Dow Chemical Company, Occupational Health, 607 Building, Midland, MI 48674, USA; E-Mail: mklee@dow.com

**Keywords:** 2,4-dichlorophenoxyacetic acid, cancer, occupation

## Abstract

Despite showing no evidence of carcinogenicity in laboratory animals, the herbicide 2,4-dichlorophenoxyacetic acid (2,4-D) has been associated with non-Hodgkin lymphoma (NHL) in some human epidemiology studies, albeit inconsistently. We matched an existing cohort of 2,4-D manufacturing employees with cancer registries in three US states resulting in 244 cancers compared to 276 expected cases. The Standardized Incidence Ratio (SIR) for the 14 NHL cases was 1.36 (95% Confidence Interval (CI) 0.74–2.29). Risk estimates were higher in the upper cumulative exposure and duration subgroups, yet not statistically significant. There were no clear patterns of NHL risk with period of hire and histology subtypes. Statistically significant results were observed for prostate cancer (SIR = 0.74, 95% CI 0.57–0.94), and “other respiratory” cancers (SIR = 3.79, 95% CI 1.22–8.84; 4 of 5 cases were mesotheliomas). Overall, we observed fewer cancer cases than expected, and a non statistically significant increase in the number of NHL cases.

## 1. Introduction

The herbicide 2,4-dichlorophenoxyacetic acid (2,4-D) is not genotoxic and has shown no evidence of carcinogenicity in laboratory animals [1–5]. However, the carcinogenicity of 2,4-D in humans has been actively debated since an early case-control report of non-Hodgkin lymphoma (NHL) and herbicide use by farmers [6]. Reviewers determined that the subsequent epidemiologic data of 2,4-D were inadequate to “conclude that any form of cancer is causally associated with 2,4-D exposure [7]”. Nonetheless, the carcinogenicity of 2,4-D continues to be examined in epidemiology studies.

Cancer mortality rates of The Dow Chemical Company 2,4-D workers in Midland, Michigan have been examined previously [8–10]. No significant excess of overall cancer mortality or NHL mortality was observed among the employees engaged in the manufacture and formulation of 2,4-D, although the number of expected deaths was small. The purpose of this study was to evaluate the cancer incidence rate in these 2,4-D workers since the last evaluation. The use of incidence data may afford greater statistical power than mortality because of the additional surviving cases, while at the same time avoiding some potential disease misclassification and treatment biases sometimes seen in mortality studies [11].

## 2. Experimental Section

The herbicide 2,4-D has been manufactured, formulated and packaged in Midland, Michigan since the 1940’s. Using the Midland cohort as reported in 2001 [10], 1316 men were identified who worked in the 2,4-D operations at any time between 1945 and 1994, and who were alive on 1 January 1985, corresponding to the initiation of the Michigan statewide cancer registry. There were only 50 eligible females in the 2,4-D manufacturing areas which were too few to provide meaningful female risk estimates. No additional work history or study subjects were added after 1994.

As in the mortality investigation of the same cohort, a relative exposure index of 0.005, 0.05, 0.5, or 5 units was assigned to each job in 2,4-D operations based on industrial hygiene monitoring and history of workplace practices [10]. Cumulative exposure for each individual was computed by summing the exposure index times job duration in years over all 2,4-D jobs held since 1945 as unit-years. Since potential for exposure had ceased before the start of follow-up for 75% of the cohort members, cumulative exposure was treated as a static variable for analysis. Cohort members were assigned to a cumulative exposure group of <1 unit-year, 1–4.99 unit years, or 5+ unit years. The same divisions were used to define cumulative duration groups. No additional exposure or work history data were added to the cohort since 1994. All jobs in the 1983 to 1994 period were in the lowest exposure intensity (very low, or 0.005), therefore all recent jobs would presumably also contribute little new exposure to the analysis.

The Michigan Department of Community Health (MDCH) ascertained incident cases by linking the cohort to the Michigan statewide cancer registry. The registry originates from the Michigan Cancer Surveillance Program of the Bureau of Local Health and Administrative Services within the MDCH. This statewide registry also incorporates data from the Metropolitan Detroit Cancer Surveillance System, a National Cancer Institute funded registry covering Wayne, Oakland and Macomb counties. The International Classification of Disease (ICD) groupings are provided in the Appendix.

We attempted to identify cancer cases in additional states. Data were linked with cases diagnosed in Arizona, a state with known deaths among former Michigan workers [12]. Of the six cases matched by this process, only one, a prostate cancer, was diagnosed prior to moving from Michigan. Since Michigan shares a border with Ohio, data were also linked to Ohio, but yielded no matches. We decided not to search in additional states because of the high cost, cumbersome regulations, and difficulty in discerning the added person years at risk [13].

The follow-up period was bounded by the latter of the study start date, 1 January 1985 and the start of work in 2,4-D, and the earlier of the date of death for decedents and the end of study date, 31 December 2007. For cancer cases, person years were allowed to accumulate beyond the diagnosis date to allow for the analysis of second primary cancers. Residency dates were additionally considered in order to accommodate eligibility requirements of the state cancer registry. All study members were considered Michigan residents while working in a Dow Michigan job. Thereafter, company address records were available only for those active at another Dow location or eligible for retirement benefits. For those members who were found to leave the state prior to 1985, follow-up was delayed until Michigan residency was resumed. Person years accrued until the end of study date, the date of death, or a permanent non-Michigan address appeared in their personnel records, whichever occurred first. A terminated employee with no address record was assumed to remain in the state following his Michigan location service. Decedents whose death certificates cited Michigan as the state of residency were determined to be eligible for study until their date of death. This was defined as Cohort 2.

In order to describe the minimum and maximum person years at risk alternative conditions were applied to create Cohorts 1 and 3. For Cohort 1, residency requirements were removed and all subjects accrued person years until the end of study or death. For Cohort 3, the most restrictive conditions were applied to halt follow-up at the date of the subject’s last known Michigan address, often earlier but never later than the Cohort 2 end of follow-up. For example, a person who retired in 1975 with a Michigan address in 1995 would have 10 person years at risk (1985 to 1995). Another person who also left employment in 1975 but with no addresses on file would be excluded from this cohort. Cancer risks were analyzed and presented for all 3 cohorts.

Standardized incidence ratios (SIR) for all cancers and specific cancers were computed using Michigan State rates for white males as the background. Statistical significance was determined using the 95% Fisher's exact confidence interval (95% CI) based on the Poisson distribution. For Cohort 2 only, parallel analyses were performed using alternative comparison areas: the SEER (Surveillance Epidemiology and End Results) registry population of Wayne, Oakland and Macomb counties, as well as a regional population of local (Midland, Saginaw and Bay) counties. Additional analyses by cumulative exposure and cumulative duration groups used Cohort 2 and state rates. Tests for trend were performed according to Breslow and Day [14].

Study conduct was reviewed and overseen by a Human Studies Review Board and the MDCH Institutional Review Board. To maintain privacy of the cancer cases, all analyses were conducted by the MDCH and no identifiable case data were released to the company investigators.

## 3. Results and Discussion

More than half of the workers were less than 50 years of age at the start of follow-up (Table 1). However, most were hired prior to the Michigan tumor registry and start of follow-up (1985). Very few racial minorities were represented in the cohort. Duration of employment in the 2,4-D operations ranged from 2 days to 34 years and cumulative exposure ranged from less than 1 to 103 units of unit-years.

Cancer incidence results and person years of follow-up for each of the 3 cohorts are presented in Table 2. The numbers of person years at risk were greatest for Cohort 1 and least for Cohort 3, corresponding to the different constraints on follow-up. By definition, the percent censored for Cohort 1 was zero. The expected numbers were at their maximum, based on Michigan cancer rates. On the other hand, the censored population was 13.5% in Cohort 3. This ultraconservative approach ended residency at the termination of employment for many workers and disqualified many others who had sparse address information. Cohorts 1 and 3 provide the upper and lower bounds for expected values. Further details are presented only for Cohort 2.

There were 244 incident cancers among the 1256 men of Cohort 2 (SIR = 0.88, 95% CI 0.78–1.00). There were 29 individuals with two independent primary cancer diagnoses and two individuals with three diagnoses each. We observed 14 cases of NHL compared to 10.27 expected (SIR = 1.36, 95% CI 0.74–2.29). The most common cancers were prostate (*n* = 62, SIR = 0.74, 95% CI 0.57–0.94), lung (*n* = 40, SIR = 0.81, 95% CI 0.58–1.11), and bladder (*n* = 22, SIR = 1.12, 95% CI = 0.70–1.70), representing more than half the cancer cases. Significantly greater than expected were 5 cases of “other respiratory” cancer compared to 1.32 expected (SIR = 3.79, 95% CI = 1.22–8.84). This category excludes cancers of the larynx, bronchus, trachea, and lung. It includes the nasal cavity, accessory sinuses, pleura, and other sites. Analyses of Cohort 2 using local and SEER comparisons differed little from the state wide comparisons.

The NHL cumulative exposure group and cumulative duration group analyses were limited by the small sample size and none was statistically significant (Table 3). Most of the subjects had worked less than a year and were in the lowest duration category. The highest SIR occurred among the workers with the longest duration. Results were similar for cumulative exposure analyses. There was little change in SIR by period of hire.

Employees engaged in the manufacture or formulation of 2,4-D experienced a decreased risk of overall cancer incidence when compared to statewide rates. There were slightly more NHL cancers than expected. We did observe some increase in risk with increasing duration and cumulative exposure, but not by decade of hire. However, these findings were based on a small number of cases and none was statistically significant. A breakdown by histological subtype revealed 7 mature B-cell lymphomas; 6 malignant lymphomas not otherwise specified; and 1 cutaneous T-cell lymphoma. No subtype was concentrated in the highest exposure category and no etiologic pattern was suggested.

In 1986, the International Agency for Cancer Research (IARC) classified the chlorophenoxy herbicides as Group 2B (possible) carcinogens [15]. This monograph evaluated the group, which in addition to 2,4-D, includes other herbicides such as 2,4,5-trichlorophenoxyacetic acid (2,4,5-T) and related impurities of polychlorinated dioxins. No human classification was made for 2,4-D. Since this review, other cohorts of phenoxy herbicide manufacturers and applicators have found no increased cancer mortality or incidence due to NHL [16–18]. A recent update of manufacturers observed a hazard ratio of 0.92 (95% CI 0.19–4.47) for deaths due to NHL in 2,4-D exposed factory workers [19]. A pooled analysis of several case-control studies also found no association between NHL and the use of 2,4-D [20]. Mechanistic and toxicological research indicates that increased cancer risk from 2,4-D exposure does not appear biologically plausible [1,2]. The findings of our study in the context of other research are not sufficient to support a relationship between 2,4-D exposure and NHL risk.

A statistically significant risk deficit for prostate cancer was observed in the manufacturing workers. Consistent with patterns in the US and Michigan, prostate cancer was the most commonly occurring cancer in the current cohort [21]. Some agricultural cohorts have indicated a positive association between prostate cancer and either 2,4-D [22], or pesticide use in general [23]. The previous mortality report of this group of manufacturing workers identified more prostate cancer deaths than expected (7 *vs.* 5) [10]. Since an industry study of triazine workers observed higher risk ratios for prostate cancer incidence than for mortality, presumably due to early screening practices, we anticipated similar results [24]. However, the SIR of 0.74 in the current study was significantly lower than expected (62 incident prostate cancers *vs.* 84 expected). It is unclear why prostate cancer is less common in the current 2,4-D cohort than the comparison populations. These results are consistent with the nested case-control study of 55,332 applicators in the Agricultural Health Study, in which no positive association of 2,4-D use and prostate cancer was reported [25]. Unlike the Agricultural Health Study, we had no information on risk factors other than age and race. Since only white males were used in the comparison rates, and there were some black males in the 2,4-D operations, any resulting bias from not including race in our study should have been toward higher rates [20].

Also unexpected was the excess risk of other respiratory cancers. Four of the five cancer cases in this category were mesotheliomas. Whereas mesothelioma is strongly associated with asbestos exposure [26], it has not been linked with 2,4-D. Notably, the Midland location has been in operation since 1897 and deaths due to mesothelioma have been previously reported at this site [12]. Because identification of the incident cases was precluded due to the confidentiality agreement with the MDCH, we were unable to scrutinize their job histories. However, it is conceivable that these four individuals were exposed to asbestos long ago in other departments at the site, in other jobs off-site, or in other non-occupational settings.

This finding illustrates a disadvantage of this occupational cohort, as well as other phenoxy herbicide cohorts, that is, the potential for co-exposures to multiple pesticides and industrial chemicals. With respect to NHL, exposures to other industrial chemicals at this site are also likely, that might increase risk [27–29]. Since the causal role of environmental risk factors for NHL is largely unknown, the contributions of other exposures are unclear [30].

Exposure was estimated by years worked in 2,4-D operations and also by a cumulative duration-intensity index. While these relative measures of exposure may not be optimal, they and the job records used in this study have several advantages over the episodic exposures reported by agricultural workers [31]. Whereas turf applicators spray fairly continuously throughout a season, exposure estimation from their job records is often inadequate [32]. In a reliability study of farmer applicators, respondents had a high percentage of agreement on ever using 2,4-D, but for more detailed questions such as *years mixed or applied* and *days per year mixed or applied*, the exact agreement was only 50 percent [33]. The current occupational cohort is strengthened by the documentation of start and stop dates and department assignments, thereby eliminating biases due to recall or observation. Further, exposures in manufacturing are likely more continuous over the course of the year than those in farm applications. Farmer applicators apply 2,4-D during a limited season which may amount to only a few days or weeks per year. Manufacturing workers may also have the opportunity for higher daily exposure. Repeated biomonitoring of German 2,4-D manufacturing workers from a single production plant between 1985 and 1989 found that most of the production workers had mean daily exposures less than 50 μg/kg/day [34]. This is in contrast to geometric mean dose levels of 2.5 μg/kg/day in US farmer applicators [35,36]. Although it is not certain that the German data are representative of all manufacturers, their exposures are well below the rodent cancer bioassay No Observable Effect Level (NOEL) of 150 mg/kg/day (the top dose tested) [37]. The large separation between worker exposures and NOEL doses in rodent toxicity studies, coupled with the lack of genotoxicity activity of 2,4-D, further supports a lack of biological plausibility for possible 2,4-D induced human cancer.

Critical to the interpretation of this study is the axiom that all cancer cases in Michigan residents appear in the Michigan registry, and that cancers among non-Michigan residents are excluded. Therefore, the main uncertainty in the study is not ascertainment, but rather determination of person years at risk. We attempted to circumscribe this uncertainty by the use of alternative follow-up definitions based on more or less constrictive use of available address data. These constraints altered the expected numbers such that the highest were in Cohort 1, the least restricted, and the lowest were in Cohort 3, the most restricted. The data for all three cohorts are presented in Table 2. In this manner the reader can observe the maximum and minimum person years at risk for this population and the related expected number of cancers for all causes. We expected that the SIRs would similarly follow so that the SIRs in Cohort 1 would be the smallest and those of Cohort 3 would be the largest, thus creating a range from lowest to highest risk in Cohorts 1, 2 and 3. This occurred for most cancer types.

One might consider Cohort 3 to be the most representative of true risk to the employees because all person years required documentation of residency. However, this conservative approach censored 14% of Michigan cancer cases, who were definitely Michigan residents at the time of diagnosis, resulting in an unexpected lowered SIR for cancers of the stomach, rectum, breast, kidney, and leukemias. The cohort was also smaller than Cohort 2 by 148 fewer men and 4457 fewer person years, further reducing the statistical power.

The construction of Cohort 2 permitted including person years between the end of Michigan employment and address changes. Nonetheless, some downward bias of the SIR may have resulted if the person years at risk were overestimated. The impact of this bias, however, must be less than the SIR as reported in Cohort 3. The higher SIRs would not change the overall conclusions. For example, the all cancer SIRs in Cohort 2 (SIR = 0.88, 244 observed *vs*. 276 expected) and Cohort 3 (SIR = 0.96, 211 observed *vs.* 220 expected) were both below the null value. The SIRs for prostate cancer were also similarly below 1.0, although the SIR in Cohort 3 was borderline significant (SIR = 0.76, 95% CI 0.57–1.00). The statistically significant SIR of 3.79 (95% CI 1.22–8.84) for other respiratory cancers in Cohort 2, was higher in Cohort 3 (SIR = 4.76) but the confidence interval was less precise (95% CI 1.53–11.11). There were 14 cases of NHL in all three Cohorts. The risk estimate for this cancer could be as high as 1.71, per Cohort 3. However, the etiology and role of 2,4-D is no more clear than the SIR of 1.36 in Cohort 2.

Because the choice of control population could also affect risk estimates, we also employed three different comparison populations, three counties, the three urban counties in the Detroit metropolitan areas defined by SEER, as well as the full state, with little resultant change in SIRs. With complete Michigan cancer ascertainment and judicious censoring of person years at risk, we found little evidence of significant bias attributable to low case ascertainment.

## 4. Conclusions

We observed no excess of overall cancer in this occupational cohort engaged in the manufacture and formulation of 2,4-D. This is consistent with the toxicology and experimental data for 2,4-D. Prostate cancer, the most common cancer in the cohort, was significantly reduced compared to background rates. Similar to the previous mortality study done at the same site, we found no statistically significant risk of NHL.

## Figures and Tables

**Table 1:** Selected characteristics of 2,4-D production workers in Cohort 2.

Age at start of follow-up	People	%
19–29	118	9.4
30–39	345	27.5
40–49	304	24.2
50–59	245	19.5
60–69	196	15.6
70+	48	3.8
Total	**1256**	
**Period of hire**		
<1960	422	33.6
1960–1985	514	40.9
>1985	320	25.5
Total	**1256**	
**Race**		
White	1208	96.2
Black	23	1.8
Hispanic	22	1.8
American Indian	3	0.2
Total	**1256**	

Person years at risk (PYAR) continued for persons diagnosed with cancer beyond the diagnosis date. If employees were known to have moved out of state, the PYAR ended at the time their address was known to be outside of Michigan.

**Table 2:** Cancer incident cases by primary site, 1985–2007 compared to Michigan White Males.

	Cohort 1				Cohort 2				Cohort 3			
	*N* = 1316, PY = 25,267				*N* = 1256, PY = 23,354				*N* = 1108, PY 18,897			

Cancer	Obs	Exp	SIR	95% CI	Obs	Exp	SIR	95% CI	Obs	Exp	SIR	95% CI
All Cancer	246	305.85	0.80	0.71–0.91	244	276.08	0.88	0.78–1.00	211	219.55	0.96	0.84–1.10
Lip, oral and pharynx	8	8.96	0.89	0.38–1.76	8	8.14	0.98	0.42–1.94	7	6.43	1.09	0.44–2.24
Stomach	5	5.21	0.96	0.31–2.24	4	4.70	0.85	0.23–2.18	3	3.74	0.80	0.16–2.34
Colon	17	23.36	0.73	0.42–1.17	17	21.03	0.81	0.47–1.29	16	16.76	0.95	0.55–1.55
Rectum	9	10.70	0.84	0.38–1.60	9	9.67	0.93	0.42–1.77	6	7.68	0.78	0.29–1.70
Pancreas	2	6.64	0.30	0.03–1.09	2	5.99	0.33	0.04–1.21	2	4.76	0.42	0.05–1.52
Other GI and Digestive	8	10.91	0.73	0.32–1.44	8	9.86	0.81	0.35–1.60	8	7.82	1.02	0.44–2.02
Larynx	5	4.96	1.01	0.32–2.35	5	4.49	1.11	0.36–2.60	4	3.55	1.13	0.30–2.88
Lung and Bronchus	41	54.51	0.75	0.54–1.02	40	49.15	0.81	0.58–1.11	36	39.04	0.92	0.65–1.28
Other Respiratory *	5	1.46	3.42	1.10–7.99	5	1.32	3.79	1.22–8.84	5	1.05	4.76	1.53–11.11
Bone and soft tissue	1	1.70	0.59	0.01–3.27	1	1.54	0.65	0.01–3.61	1	1.24	0.81	0.01–4.49
Melanoma of skin	9	9.34	0.96	0.44–1.83	9	8.50	1.06	0.48–2.01	8	6.77	1.18	0.51–2.33
Breast	1	0.66	1.52	0.02–8.43	1	0.60	1.67	0.02–9.27	0	0.47	0	
Prostate gland	62	93.44	0.66	0.51–0.85	62	84.11	0.74	0.57–0.94	51	66.86	0.76	0.57–1.00
Other genital *	3	1.87	1.60	0.32–4.69	3	1.74	1.72	0.35–5.04	3	1.44	2.08	0.42–6.09
Urinary bladder	22	21.73	1.01	0.63–1.53	22	19.57	1.12	0.70–1.70	19	15.59	1.22	0.73–1.90
Kidney and renal pelvis	8	9.14	0.88	0.38–1.72	8	8.29	0.97	0.42–1.90	5	6.57	0.76	0.25–1.78
Other urinary *	3	0.93	3.23	0.65–9.43	3	0.84	3.57	0.72–10.44	3	0.67	4.48	0.90–13.08
Brain and other CNS	3	3.78	0.79	0.16–2.32	3	3.43	0.87	0.17–2.54	3	2.75	1.09	0.22–3.19
Hodgkin’s disease	1	1.03	0.97	0.01–5.40	1	0.95	1.05	0.01–5.86	1	0.77	1.30	0.02–7.23
Non-Hodgkin lymphoma	14	11.34	1.23	0.67–2.07	14	10.27	1.36	0.74–2.29	14	8.18	1.71	0.93–2.87
Multiple myeloma	2	3.52	0.57	0.06–2.05	2	3.17	0.63	0.07–2.28	2	2.52	0.79	0.09–2.87
Leukemias	7	8.04	0.87	0.35–1.79	7	7.25	0.96	0.39–1.98	5	5.79	0.86	0.28–2.02
All other	10	12.63	0.79	0.38–1.46	10	11.42	0.88	0.42–1.61	9	9.11	0.99	0.45–1.88

Cohort 1 assumes the entire cohort remained in Michigan through December 31, 2007; For Cohort 2, person years at risk (PYAR) continued for persons diagnosed with cancer beyond the diagnosis date. If employees were known to have moved out of state, the PYAR ended at the time their address was known to be outside of Michigan; Cohort 3 was restricted to employees considered to be Michigan residents for the entire period;

* Other Respiratory include 1 carcinoma of the sinuses, 4 mesothelioma; Other Genital include 1 penis, 2 testis; Other Urinary include 1 urethra, 2 ureter.

**Table 3:** Observed and expected numbers of NHL by groups, Cohort 2, 1985–2007.

Exposure measure	People	Observed	Expected	SIR	95% CI
**Duration (years)**					
<1	770	7	6.50	1.08	0.43–2.22
1–4.99	338	3	2.47	1.21	0.25–3.55
5+	148	4	1.30	3.08 *	0.84–7.88
**Intensity x duration** (exposure years)					
<1	1001	9	7.25	1.24	0.57–2.36
1–4.99	149	2	1.63	1.23	0.15–4.43
5+	106	3	1.39	2.16 *	0.45–6.31
**Decade of Hire**					
1945–1949	181	4	2.54	1.57	0.43–4.03
1950–1959	241	4	3.06	1.31	0.36–3.35
1960–1969	305	4	2.25	1.78	0.48–4.56
1970–1979	255	2	1.47	1.36	0.17–4.92
1980–1989	167	0	0.69	0.00	0.00–5.35
1990–1994	107	0	0.26	0.00*	0.00–14.19

* *P* test for trend for duration (*p* = 0.12), exposure years (*p* = 0.46) and decade of hire (*p* = 0.43); Person years at risk (PYAR) continued for persons diagnosed with cancer beyond the diagnosis date. If employees were known to have moved out of state, the PYAR ended at the time their address was considered to be outside of Michigan.
